# S. E. Texas Medical Society Revived

**Published:** 1892-04

**Authors:** 


### SOUTHEAST TEXAS MEDICAL ASSOCIATION REVIVED.

BEAUMONT, TEXAS, March 30th, 1892.

A meeting of physicians was held in the parlors of the Crosby house last evening for the purpose of reviving the old Southeast Texas Medical Society.

The object of the meeting being briefly stated, the following temporary officers were elected. B. F. Calhoun, Chairman, and F. Hadra, Secretary. The organization of the society was effected by the election of the following permanent officers:

President, Dr. A. N. Perkins; First Vice-President, Dr. C. Y. Thompson; Second Vice-President, Dr. S. W. Sholars; Secretary and Treasurer, Dr. F. Hadra.

The constitution and by-laws of the old Southeast Texas Medical Society were read and adopted, with the exception of fee bills, which were eliminated. Beaumont was made the permanent meeting place of the society, and the second Tuesday of January, April, July and October the time of meeting.

Drs. J. Saunders, B. F. Calhoun and J. B. Roberts were elected censors.

The following delegates to the State Medical Association were appointed: Drs. Calhoun, Sholars, Roberts, Blewitt and Price, with Drs. Thompson, Callen, Saunders, Seastrunk, Cruse and Ogden alternates. The President and Secretary were added as regular delegates.

Drs. Saunders, Price and Roberts were appointed to read papers, respectively, at the next meeting, the discussion to be led by Drs. Calhoun, Sholars and Thompson in the order named.

